# Prevalence of *Toxocara* and *Toxascaris* infection among human and animals in Iran with meta-analysis approach

**DOI:** 10.1186/s12879-020-4759-8

**Published:** 2020-01-07

**Authors:** Aida Vafae Eslahi, Milad Badri, Ali Khorshidi, Hamidreza Majidiani, Elham Hooshmand, Hamid Hosseini, Ali Taghipour, Masoud Foroutan, Nader Pestehchian, Farzaneh Firoozeh, Seyed Mohammad Riahi, Mohammad Zibaei

**Affiliations:** 1 Department of Parasitology, Faculty of Medical Sciences, Tarbiat Modares University, Tehran, Iran; 2 Department of Epidemiology, School of Medicine, Ilam University of Medical Sciences, Ilam, Iran; 3 Department of Parasitology, Faculty of Veterinary Medicine, Rasht Branch, Islamic Azad University, Rasht, Iran; 4 Department of Parasitology and Mycology, School of Medicine, Alborz University of Medical Sciences, 3149779453, Karaj, Iran; 5 Abadan Faculty of Medical Sciences, Abadan, Iran; 6 Department of Parasitology and Mycology, School of Medicine, Isfahan University of Medical Sciences, Isfahan, Iran; 7 Infectious Diseases and Tropical Medicine Research Center, Isfahan University of Medical Sciences, Isfahan, Iran; 8 Department of Microbiology, School of Medicine, Alborz University of Medical Sciences, Karaj, Iran; 9 Department of Epidemiology and Biostatistics, School of Health, Birjand University of Medical Sciences, Birjand, Iran; 10 Evidence-based Phytotherapy and Complementary Medicine Research Center, Alborz University of Medical Sciences, Karaj, Iran

**Keywords:** Systematic review, Meta-analysis, Toxocariasis, *Toxocara canis*, *Toxocara cati*, *Toxascaris leonina*, Iran

## Abstract

**Background:**

Toxocariasis is a worldwide zoonotic parasitic disease caused by species of *Toxocara* and *Toxascaris*, common in dogs and cats. Herein, a meta-analysis was contrived to assess the prevalence of *Toxocara*/*Toxascaris* in carnivore and human hosts in different regions of Iran from April 1969 to June 2019.

**Methods:**

The available online articles of English (PubMed, Science Direct, Scopus, and Ovid) and Persian (SID, Iran Medex, Magiran, and Iran Doc) databases and also the articles that presented in held parasitology congresses of Iran were involved.

**Results:**

The weighted prevalence of *Toxocara/Toxascaris* in dogs (*Canis familiaris*) and cats (*Felis catus*) was 24.2% (95% CI: 18.0–31.0%) and 32.6% (95% CI: 22.6–43.4%), respectively. Also, pooled prevalence in jackal (*Canis aureus*) and red fox (*Vulpes vulpes*) was 23.3% (95% CI: 7.7–43.2%) and 69.4% (95% CI: 60.3–77.8%), correspondingly. Weighted mean prevalence of human cases with overall 28 records was 9.3% (95% CI: 6.3–13.1%). The weighted prevalence of *Toxocara canis*, *Toxocara cati*, and *Toxascaris leonina* was represented as 13.8% (95% CI: 9.8–18.3%), 28.5% (95% CI: 20–37.7%) and 14.3% (95% CI: 8.1–22.0%), respectively.

**Conclusion:**

Our meta-analysis results illustrate a considerable prevalence rate of *Toxocara/Toxascaris*, particularly in cats and dogs of northern parts of Iran. The presence of suitable animal hosts, optimum climate and close contact of humans and animals would have been the reason for higher seroprevalence rates of human cases in our region. Given the significance clinical outcomes of human *Toxocara/Toxascaris*, necessary measures should be taken.

## Background

Zoonoses are those complications which are transmissible between human and animal populations [1]. In this regard dogs and cats are considered as a public health concern, as they may harbor various pathogens such as zoonotic helminths including *Toxocara* species [2]. Toxocariasis is a worldwide parasitic infection, primarily rendered by *T. canis* in dogs, *T. cati* in cats and foxes and *T. leonina* in a wide range of carnivores [3]. Mature worms lay eggs in the intestinal lumen of their host, which are excreted into the environment via defecation and pass their developmental stages in optimum soil and climate conditions. Upon ingestion of embryonated eggs by another host, the larvae would emerge and invade the intestinal mucosa, then migrate through viscera such as lungs, liver, and kidneys. In addition, transplacental and transmammary transmission to puppies and kittens are important routes of infection. In an epidemiological perspective, animal hosts parasitized by adult worms in their gut can disseminate infection by shedding parasite eggs into environment [4]. In an epidemiological perspective, animal hosts parasitized by adult worms in their gut, can shed parasite eggs, hence considered as a source for dissemination of the infection [5]. Human infection occurs by accidental ingestion of eggs, and, to a lesser extent, via pica and devouring on the paratenic hosts, including chicken, cattle, lamb, pig, and earthworms [4, 6]. Consequently, developmentally-arrested larvae migrate through body organs, but don’t develop into mature worms; hence, they provoke an array of syndromes enclosing VLM, NLM, and OLM as well as covert infection and asymptomatic toxocariasis [7–9]. Although rare, cardiac-associated toxocariasis is a serious, life-threatening complication due to VLM which has recently been emphasized [10].

Most of the infected individuals manifest nonspecific symptoms such as a cough, rhonchus, dyspnea and pyrexia along with hepatomegaly and eosinophilic granuloma, which implicates diagnosis of the infection using more sensitive approaches such as immunological assays i.e., ELISA for screening and Western blot for confirmation, rather than histological or parasitological methods [4, 11].

Toxocariasis cause by *T. cati* and *T. canis* frequently impacts young cats and dogs from birth to 1 year old, entailing respiratory signs (coughing due to pulmonary larval migration), general failure to thrive (retarded growth, emaciation, debilitated body coat and arthralgia) and intestinal disorders (alternating diarrhea and constipation, pot-belly and vomiting). No remarkable trait is seen following *Toxascaris* infection in dogs and/or cats and it is usually well-tolerated [3–5].

One of the characteristic of helminthic parasites is the stimulation of the immune system that leads to increased Th2 response and high production of IL-4, IL-5, IL-9, IL-10, IL-13, eosinophils, and IgE. *Toxocara* larvae can causes severe hyper eosinophilia and allergic involvements with effect on IgE and IL-5. Consequently, the production of specific antibodies provides the most complete evidence for *Toxocara* infection, which is the base of diagnostic tests such as ELISA and Western blot for reactivity to larval TES antigen [11–13].

Iran, a Middle Eastern country, possesses several climatological areas with particular characteristics in each region; this would have a significant bias on the epidemiology of *Toxocara/Toxascaris* species. In the previous studies the infection of dogs and cats with *Toxocara* species in different parts of Iran has been shown [14]. Despite the prevalence of *Toxocara canis* in the most areas, molecular studies on cat nematodes in Shiraz, in south-central Iran showed that, the most prevalent one is *T. cati* [15]. *Toxocara vitulorum* is frequently found in ruminants. Its main hosts are cattle and buffalo in tropical and sub-tropical regions [16]. It has been reported that 16% (95% CI: 11–21%; 470 out of 3031 samples) of soil samples gathered from public parks of the Iran were positive for *Toxocara* spp. eggs [17].

On the other hand, due to increasing body of work on *Toxocara* prevalence in various human/animal hosts in Iran, a comprehensive review would be exceedingly beneficial for appraising progresses about this zoonosis. Therefore, this meta-analysis attempts to fill the current gaps and provides insights into parasite prevalence with respect to host type, *Toxocara* and *Toxascaris* species, and geographical region in the country.

## Methods

### Study area

Iran has a population of approximately 80 million (as of 2015), and is located between 25°3ʹ and 39°47ʹN and 44°5ʹ and 63°18ʹE, which covers a wide territory in the Middle East area (1,648,195 km2). The country borders Afghanistan and Pakistan to the east, Iraq and Turkey to the west, the Persian Gulf and Oman Sea to the south, and Azerbaijan, Armenia, and Turkmenistan to the north. The Iranian plateau climate is generally hot and dry, however the Caspian Sea coast in northern parts, comprising Golestan, Mazandaran and Guilan provinces, is Mediterranean-like, demonstrating heavy rainfalls, vegetation-enriched, surrounded by dense forests and a diverse range of carnivorous animals These geo-ecological features would provide a well-established setting for most parasites, e.g. soil-transmitted helminthiases, to localize in the area and parasitize many canid species. Also, the country is a mountainous region with several mountain ranges, mostly located at the western and northern parts such as Zagros mountain ranges with colder winters and heavy snowfalls. The annual precipitation is 680 mm in the eastern part of the plain and more than 1700 mm in the western parts [18–21].

### Search strategy

The PRISMA protocol (preferred reporting items for systematic reviews and meta-analysis) was employed to conduct this meta-analysis [22]. In order to assess the prevalence of *T. canis*, *T. cati* and *T. leonina* in humans and carnivores of different parts of Iran, we investigated the available online articles of both Persian (SID, Iran Medex, Magiran, Iran Doc) and English (PubMed, Science Direct, Scopus, Ovid) databases. The search include between April 1969 and June 2019. Also, the articles that presented in held Parasitology congresses of Iran were involved. A combination of the following search terms were employed in our literature searches as follows: (“Toxocariasis” OR “*Toxocara* infection” OR “*Toxocara canis*” OR “*Toxocara cati*” OR “*Toxascaris leonina*”) AND (“Carnivora” OR “Human”) AND (“Prevalence” OR “Epidemiology”) AND (“Iran”).

### Study selection and data extraction

After hand searching in bibliographic list of obtained full-text records for any related literature as well as removing duplicates, two independent reviewers screened the titles and abstracts for initial inclusion. A third reviewer was also involved for consensus in the case of any disagreements. Finally, those records that met the following inclusion criteria were eligible to enter our meta-analysis: (A) Peer-reviewed originally-published papers both in English or Persian; (B) Being available online between April 1969 till June 2019; (C) Cross-sectional investigations that assessed the prevalence of *Toxocara* spp. in various carnivores and human populations in Iran; (D) Studies that detected *Toxocara* infection using at least one of the parasitological, serological and molecular methods; (e) exact total sample size, positive samples and the respective prevalence rates were available. Empirical studies and any kind of review papers were excluded and failed for further analysis. A detailed variable of each of articles, including: province, year of publication, study design, sample size, detection method, and prevalence rates, in addition to animal species and sampling method for animal-based investigations were gathered. In this study, the JBI critical appraisal checklist for prevalence studies was employed [23].

### Study quality assessment

The JBI checklist was used for quality assessment of the included articles. This checklist contains eight questions with four options including, Yes, No, Unclear, and Not applicable (Additional file 1: Figure S1). Briefly, a study can be awarded a maximum of one star for each numbered item. The papers with a total score of ≤6 and ≥ 7 points were specified as the moderate and high quality, respectively. Based on the obtained score, the authors have decided to include and exclude the papers [23].

### Meta-analysis

Briefly, meta-analysis was yielded as a forest plot representing the prevalence estimates and related confidence intervals of each study along with summary measures. Also, the heterogeneity was analyzed using STATA statistical software (Version 8.2) to calculate Cochran’s Q and I^2^ statistics. I^2^ values of 25, 50, and 75% were considered as low, moderate and high heterogeneity, respectively [24]. Furthermore, the funnel plot based on Egger’s regression test illustrates publication bias and small study effects. In the current study, I^2^ was substantial; therefore, we used a random effects model at a 95% CI, to give a more conservative estimate of the *Toxocara* infection prevalence.

## Results

### Hosts

**Fig. 1:**
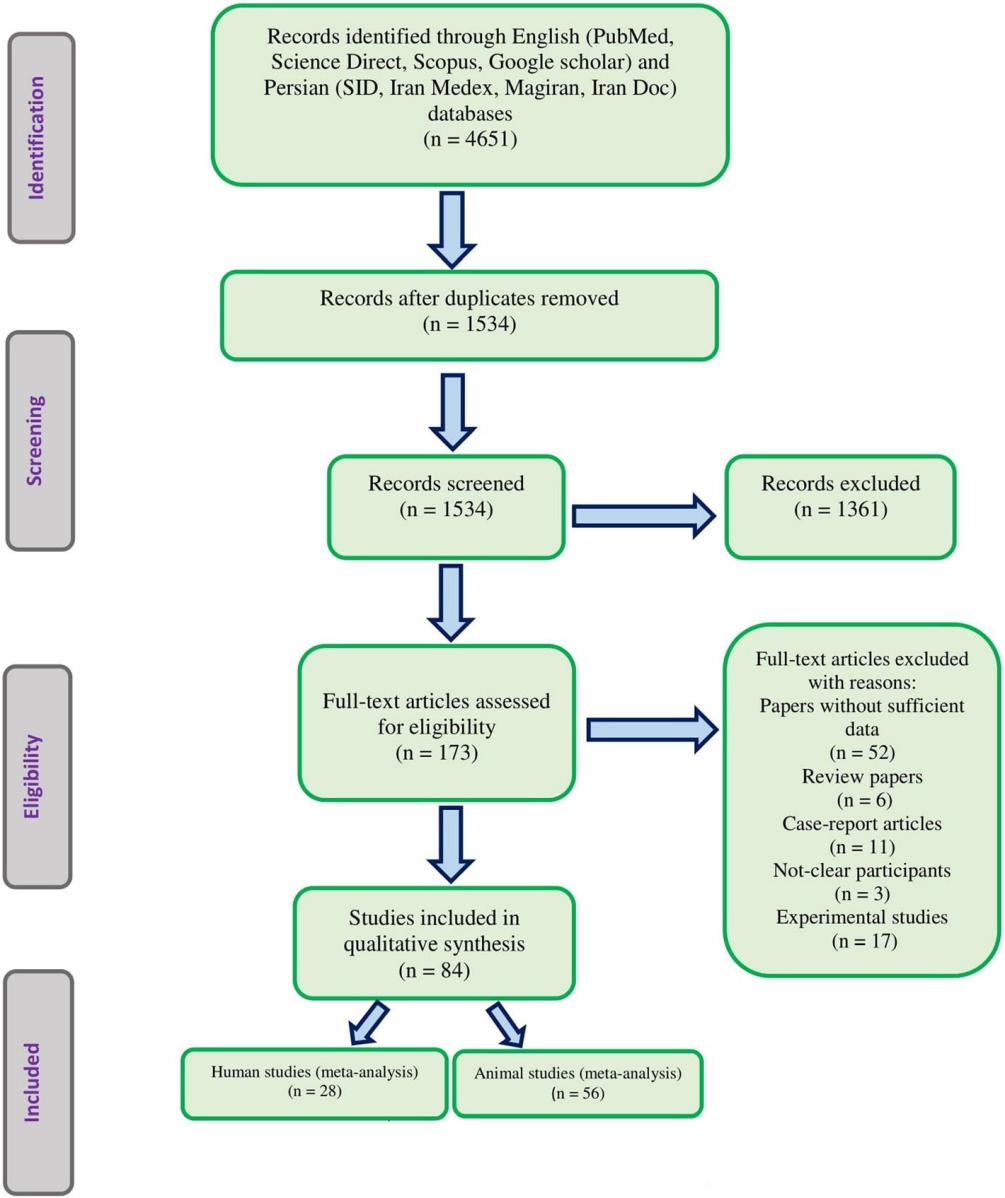
Flowchart describing the study design process

Following systematic search of eight databases, totally 28 records human studies and 56 animal investigations were found eligible regarding *Toxocara*/*Toxascaris* (Fig. 1). During a 19-years period, 11,781 human individuals were examined and the calculated weighted prevalence was 9.3% (95% CI: 6.1–13.1%) (Tables 1 and 2). The trend line of human *Toxocara*/*Toxascaris* infection demonstrated that the prevalence has declined in spite of increased bulk of work on human population (Additional file 2: Figure S2). Most records (10 studies) were conducted in both rural and urban circumstances, however seroprevalence was mostly predominant in urban regions with 14% (95% CI: 5.6–25.3%) (No showed data). People under 20 years old were mostly examined by serodiagnosis approach, indicating 8.2% (95% CI: 4.6–12.7%) seroprevalence rate (Additional file 3: Figure S3).

**Table 1:** Pooled prevalence of *Toxocara* infection among human and animals in Iran

*Toxocara/Toxascaris*	# of study	Total sample size	Positive	Pooled prevalence (95% CI)	Q	df	I^2^
Human part							
National Prevalence	28	11,377	1367	9.3 (6.1–13.1)	1114.7	27	97.6
Quality grade							
high	18	7597	812	10.0 (5.9–15.0)	743.7	17	97.7
moderate	10	3780	555	8.1 (3.3–14.7)	322.3	9	97.2
Publication Year							
2000–2005	2	583	146	24.9 (21.4–28.5)	1114.1	1	99.9
2005–2010	5	2939	391	9.9 (2.3–21.6)	285.9	4	98.6
2010–2015	8	1477	165	8.6 (1.9–19.3)	240.3	7	97.1
2015–2019	13	6378	665	8.0 (4.2–12.7)	471.5	12	97.5
Animal part							
Prevalence in Dog	29	4065	844	24.2 (18.0–31.0)	100.1	28	95.2
Prevalence in Cat	20	1670	511	32.6 (22.6–43.4)	386.9	19	95.1
Prevalence in Jackal	4	57	13	23.3 (7.7–43.2)	6.9	3	56.5
Prevalence in Red fox	2	111	76	69.4 (60.3–77.8)	–	–	–
Wild cat	1	8	5	62.5 (24.5–91.5)	–	–	–
Parasite species in carnivores							
*T. canis* (overall)	31	4420	545	13.8 (9.8–18.3)	449.2	30	93.3
Publication Year							
> 2005	4	368	17	5.0 (0.6–12.5)	19.1	3	84.3
2005–2010	6	638	159	26.7 (12.8–43.3)	95.9	5	94.8
2010–2015	14	2638	290	13.1 (7.8–19.4)	204.7	13	93.6
2015–2019	7	776	79	11.0 (6.2–16.9)	27.1	6	77.8
*T. cati* (overall)	24	1811	503	28.5 (20.0–37.7)	394.7	23	94.2
Publication Year							
> 2005	3	221	95	45.3 (26.8–64.4)	11.2	2	82.1
2005–2010	10	792	230	28.6 (18.0–40.6)	112.1	9	92.0
2010–2015	7	520	98	21.6 (8.0–39.3)	112.3	6	94.7
2015–2019	4	278	80	28.9 (3.0–66.2)	105.2	3	97.1
*T. leonina* (overall)	20	3150	420	14.3 (8.1–22.0)	498.4	19	96.2
Publication Year							
> 2005	2	220	81	36.0 (29.7–42.5)	498.4	1	99.8
2005–2010	3	329	48	12.1 (0.8–32.5)	42.4	2	95.3
2010–2015	11	2032	175	12.2 (4.6–22.6)	253.0	10	96.0
2015–2019	4	562	98	13.4 (4.9–25.0)	30.4	3	90.1

**Table 2:** Baseline characteristics of included studies for human toxocariasis in Iran

Author(s)	Country	Publication year	Sample size	Dm^a^			P^e^ [N (%)]	SM^f^		
				P^b^	S^c^	M^d^		ELISA^g^	WB^h^	IFAT^i^
S. M. Sadjjadi	Shiraz	2000	519		*		133 (25.60)	*		
H. Yousefi	Chaharmahal-Va Bakhtiari	2001	64		*		13 (20.31)			*
L. Akhlaghi	Kermanshah	2006	260		*		22 (8.46)	*		
M. Fallah	Hamadan	2007	544		*		29 (5.30)	*		
A. Nourian	Zanjan	2007	810		*		22 (2.70)	*		
S. M. Alavi	Khuzestan	2008	115		*		16 (13.9)	*		
M. Sharif	Mazandaran	2010	1210		*		302 (25.00)	*		
S. M. Alavi	Khuzestan	2011	203		*		4 (2.00)	*		
Kh. Agin	Tehran	2012	89		*		14 (16.00)	*		
M. Zibaei	Lorestan	2013	85		*		3 (3.50)	*	*	
Y. Gharedaghi	East Azerbaijan	2014	336		*		99 (29.46)	*		
Sh. Khademvatan	Khuzestan	2014	95		*		4 (4.30)	*	*	
M. Zibaei	Shiraz	2015	98		*		33 (33.67)	*	*	
A. Hosseini Safa	Isfahan	2015	427		*		6 (1.39)	*		
S. Allahdin	Khuzestan	2015	144		*		2 (1.38)	*	*	
F. Berenji	Khorasan	2016	93		*		1 (1.07)	*		
T. Momeni	West Azerbaijan	2016	397		*		12 (3.00)	*		
M. Kh. Shahraki	Sistan and Baluchestan	2017	364		*		14 (3.8)	*		
H. Mahmoudvand	Lorestan	2018	316		*		14 (4.40)	*		
S. Shokouhi	Ilam	2018	383		*		84 (22.00)	*		
M. Beiromvand	Khuzestan	2018	400		*		11 (2.70)	*		
Z. Baghani	Tehran	2018	374		*		21 (5.60)	*		
S. Khoshnood	Ilam	2018	300		*		35 (11.70)	*		
S. Ashtari	Urmia	2018	1002		*		172 (17.22)	*		
B. Sarkari	Shiraz	2018	617		*		39 (6.30)	*		
S. Aghamolaie	Mazandaran	2018	630		*		148 (23.50)	*		
V. Raissi	Ilam	2018	539		*		97 (17.99)	*		
M. K. Shahraki	Sistan and Baluchestan	2019	963		*		17 (1.70)	*		

^a^Detection method, ^b^Parasitology, ^c^Serology, ^d^Molecular, ^e^Prevalence, ^f^Serological method, ^g^Enzyme-linked immunosorbent assay, ^h^Western blot, ^i^Indirect fluorescent antibody test

A number of 29 entries contributed to prevalence of *Toxocara*/*Toxascaris* in dogs (*Canis familiaris*), showing a prevalence of 24.2% (95% CI: 18.0–31.0%). The weighted prevalence of *Toxocara*/*Toxascaris* was higher in 20 investigations which examined cats (*Felis catus*) [32.6% (95% CI: 22.6–43.4%)] (Tables 1 and 3). Interestingly, one study also used serodiagnosis in cats indicating a 53.8% (95% CI: 39.5–67.8%) seroprevalence (Additional file 4: Figure S4).

**Table 3:** Baseline characteristics of included studies for animal toxocariasis in Iran

Author(s)	Area	Publication year	Sample size	Animals					Sampling		Dm^a^			P^*e*^ [N (%)]								
				Dog	Cat	Jackal	Wild cat	Red fox	Feces	Biopsy	P^b^	S^c^	M^d^	Parasite (species)				Animals				
														*T. canis*	*T. cati*	*T. leonina*	Overall	Dog	Cat	Jackal	Wild cat	Red fox
A. Sadighian	Shahsavar	1969	43	23		20				*	*			10 (23.2)				8 (34.7)		2 (10.0)		
A. Sadighian	Shahsavar	1970	8				8			*	*				5 (62.5)						5 (62.5)	
A. Mirzayans	Tehran	1971	105		105					*	*			2 (1.9)	33 (31.4)				35 (33.3)			
A. Nourian	Zanjan	1998	115	115						*	*			2 (1.7)		60 (52.2)		62 (53.9)				
S. M. Sadjjadi	Shiraz	2001	108		108					*	*				57 (52.8)				57 (52.8)			
D. Mehrabani	Shiraz	2002	105	105						*	*			3 (2.9)		21 (20.0)		24 (22.8)				
Gh. R. Razmi	Khorasan	2006	100	100						*	*			39 (39.0)		7 (7.0)		46 (46.0)				
A. Dalimi	Western Iran	2006	115	83		10		22		*	*			7 (6.1)		37 (32.2)		32 (38.5)		4 (40.0)		8 (36.3)
E. Changizi	Guilan	2007	50		50					*	*				4 (8.0)				4 (8.0)			
M. Sharif	Mazandaran	2007	100		100					*	*				44 (44.0)				44 (44.0)			
M. Zibaei	Shiraz	2007	114		114					*	*				26 (42.6)	4 (12.9)			30 (26.3)			
M. Arbabi	Kashan	2009	113		113					*	*				15 (13.3)				15 (13.3)			
A. Daryani	Mazandaran	2009	50	50					*	*	*			30 (60.0)				30 (60.0)				
M. Esmaeilzadeh	Zanjan	2009	100		100					*	*				8 (8.0)				8 (8.0)			
Gh. R. Razmi	Khorasan Razavi	2009	174	174					*		*			20 (11.5)				20 (11.5)				
A. Eslami	Semnan	2010	50	50						*	*			11 (22.0)				11 (22.0)				
B. Meshgi	Tehran	2010	55		55					*	*				29 (52.7)				29 (52.7)			
B. Meshgi	Tehran	2010	55		55				*						22 (40.0)				22 (40.0)			
B. Meshgi	Tehran	2010	55		52							*			28 (53.8)				28 (53.8)			
M. Sharif	Mazandaran	2010	100		100					*	*				44 (44.0)				44 (44.0)			
Sh. Shirazi	East Azerbaijan	2010	50		50					*	*				10 (20.0)				10 (20.0)			
M. Zare-Bidaki	Ardabil	2010	149	59		1		89		*	*			52 (34.9)			39 (26.2)	23 (38.9)				68 (76.4)
H. Borji	Khorasan	2011	52		52					*	*				15 (28.8)	4 (7.6)						
Y. Garedaghi	East Azerbaijan	2011	100	100					*		*			12 (12.0)				12 (12.0)				
Sh. Gholami	Mazandaran	2011	50	50						*	*			30 (60.0)		1 (2.0)						
M. Beiromvand	Khorasan Razavi	2012	77	77					*		*					22 (29.0)	19 (25.0)	41 (53.2)				
M. Mirzaei	Kerman	2012	100	100					*		*			10 (10.0)				10 (10.0)				
M. Mirzaei	Kerman	2012	70	70					*		*			3 (4.3)		1 (1.4.0)		4 (5.7)				
N. Pestechian	Isfahan	2012	96	96						*	*			6 (6.25)		21 (21.9)						
A. Pezeshki	Tehran	2013	138		138				*		*				13 (9.4)				13 (9.4)			
A. Adinezadeh	Khorasan Razavi	2013	100	100						*	*			7 (7.0)		53 (53.0)		60 (60.0)				
F. Mikaeili	Shiraz	2013	30		30					*	*		*		8 (26.7)				8 (26.7)			
M. Mirzaei	Kerman	2013	100	100					*		*			9 (9.0)		1 (1.0)		10 (10.0)				
F. Parsa	Lorestan	2014	80	80						*	*			8 (10.0)		14 (17.7)		22 (27.5)				
Y. Garedaghi	East Azerbaijan	2014	125	125					*		*			4 (3.2)				4 (3.2)				
M. Yakhchali	West Azerbaijan	2014	150	150					*		*			47 (31.3)				47 (31.3)				
Sh. Sarvi	Mazandaran	2014	100	100					*		*			27 (27.0)		4 (4.0)		31 (31.0)				
J. Gharekhani	Hamadan	2014	210	210					*		*			4 (1.9)				4 (1.9)				
Sh. Khademvatan	Khuzestan	2014	140		140				*		*						63 (45.0)		63 (45.0)			
Sh. Sarvi	Mazandaran	2014	100	100					*		*				27 (27.0)				27 (27.0)			
A. A. Shokri	East Azerbaijan	2015	50		50				*		*				4 (8.0)				4 (8.0)			
Y. Garedaghi	East Azerbaijan	2015	100		100				*		*				12 (12.0)				12 (12.0)			
S. R. Emampour	Khorasan	2015	100	100						*	*			29 (29.0)	7 (7.7)			36 (36.0)				
N. Hajipour	East Azerbaijan	2015	50		50					*	*				39 (78.0)	15 (30.0)						
K. Sardarian	Hamadan	2015	1257	1257					*		*			94 (6.3)		39 (2.6)		133 (10.5)				
K. Arzamani	Khorasan	2016	32	32						*	*			3 (9.3)				3 (9.3)				
F. Mirani	Kermanshah	2016	138	138					*		*			24 (17.4)				24 (17.4)				
A. Geraili	Sistan and Baluchestan	2016	30	30						*	*			7 (23.3)	1 (3.3)			8 (26.6)				
S. Torkan	Isfahan	2017	147		147				*		*		*		26 (17.7)				26 (17.7)			
M. Yakhchali	East Azerbaijan	2017	51		51					*	*				44 (86.3)	6 (11.8)			50 (98.0)			
A. V. Eslahi	Guilan	2017	50	27	12	11				*	*			8 (16.0)	9 (18.0)	6 (12.0)		9 (33.3)	9 (75.0)	5 (45.4)		
M. Beiromvand	Khuzestan	2018	167	167					*				*	5 (3.0)		11 (6.6)		25 (15.01)				
A. Amouei	Mazandaran	2018	58	42		16				*	*			6 (10.3)				4 (9.5)		2 (12.5)		
M. A. Mohaghegh	Kermanshah	2018	301	301					*		*			26 (8.6)		75 (2.9)		101 (33.5)				

^a^Detection method, ^b^parasitology, ^c^serology, ^d^molecular, ^e^prevalence

Four studies (all necropsy-based) dedicated to prevalence of *Toxocara*/*Toxascaris* in jackal (*Canis aureus*), representing a 23.3% (95% CI: 7.7–43.2%) frequency. A high prevalence among examined carnivores in Iran was observed in two studies of red fox (*Vulpes vulpes*) with 69.4% (95% CI: 60.3–77.8%) and one study of wildcat (*Felis silvestris*) with 62.5% (95% CI: 24.5–91.5%) (Tables 1 and 3).

According to the detection method, the highest total prevalence of *T. canis* in feces was related to the formalin-ether method [10.5% (95% CI: 5.8–16.3%)] (Additional file 5: Figure S5). Also the most total prevalence of *T. cati* in feces was related to the formalin-ether method [13.4% (95% CI: 9.7–17.7%)] (Additional file 6: Figure S6).

### Parasite species

**Fig. 2:**
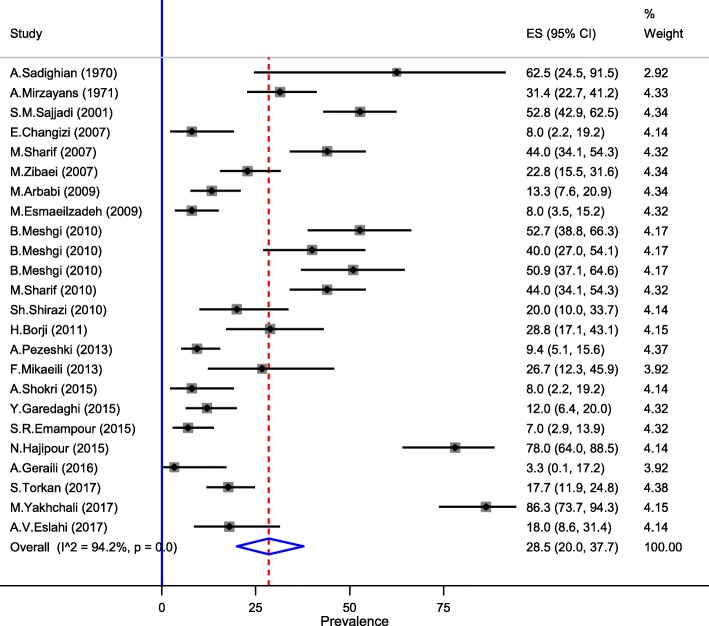
The total prevalence of *T. cati* infection in carnivores of Iran

**Fig. 3:**
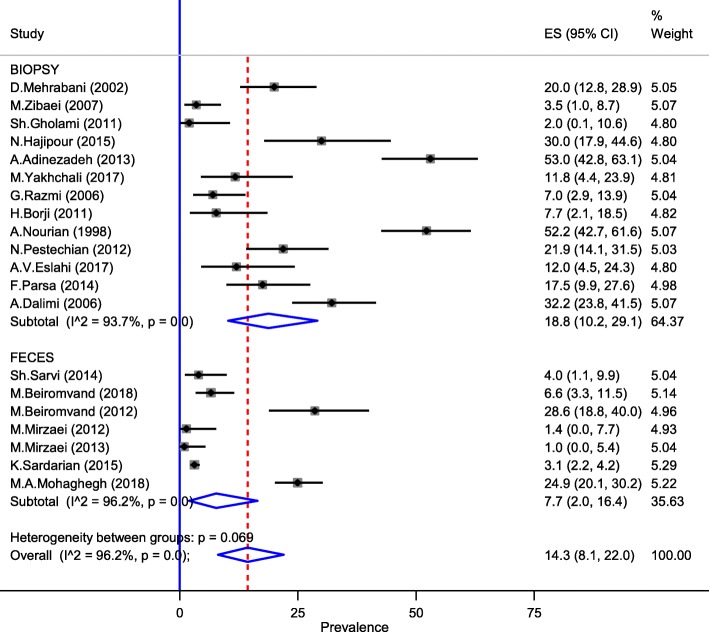
The weighted prevalence of *T. leonina* in Iran carnivores by study method

**Fig. 4:**
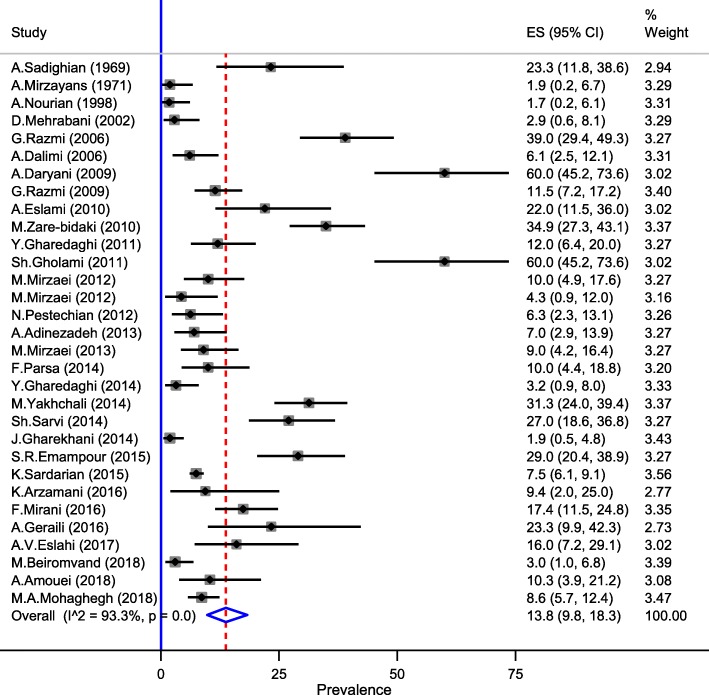
The total prevalence of *T. canis* infection in carnivores of Iran

**Fig. 5:**
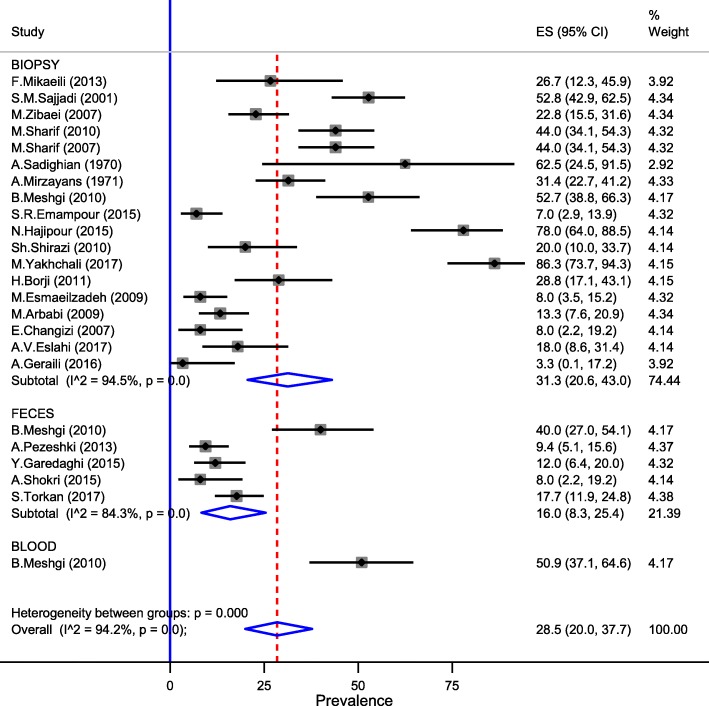
The weighted prevalence of *T. cati* in Iran carnivores by study method

**Fig. 6:**
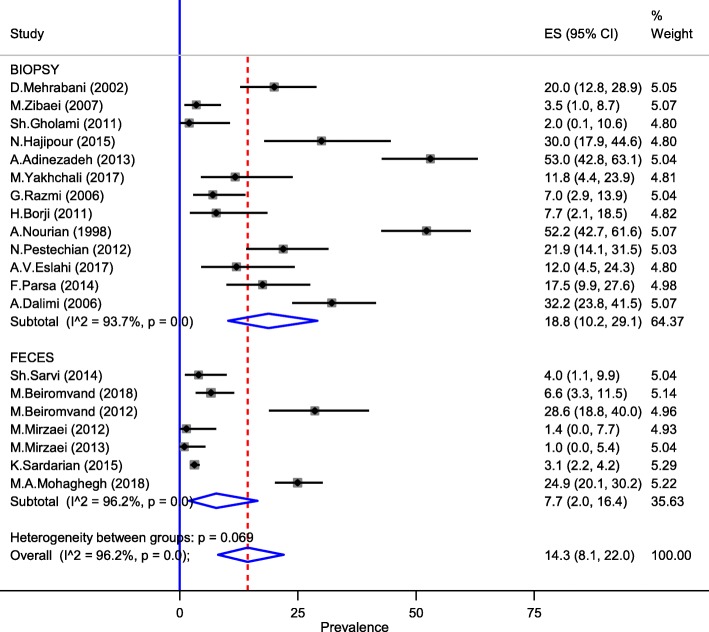
The weighted prevalence of *T. leonina* in Iran carnivores by study method

**Fig. 7:**
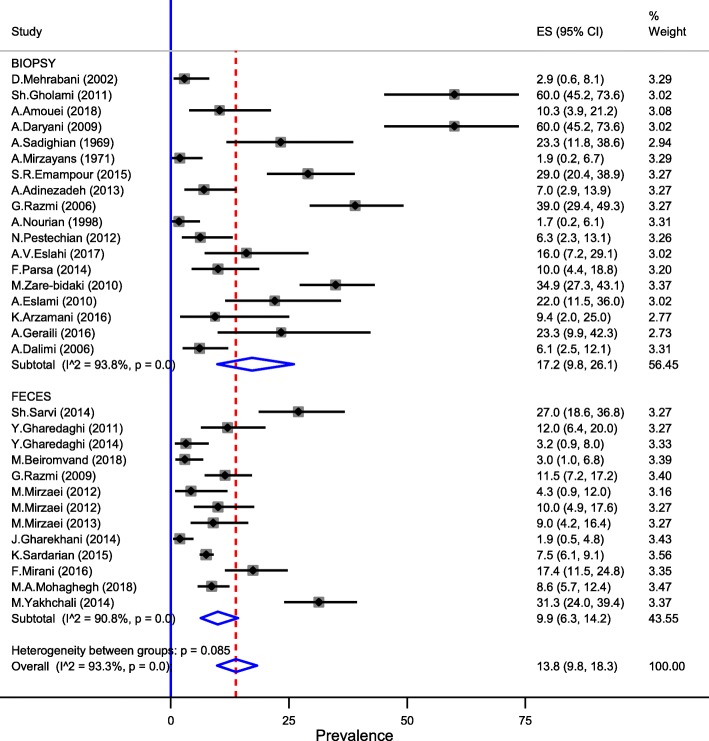
The weighted prevalence of *T.canis* in Iran carnivores by study method

Among *Toxocara*/*Toxascaris* species examined through included studies in Iran, *T. cati* possessed the highest prevalence rate with 28.5% (95% CI: 20.0–37.7%) (25 records), whereas the pooled prevalence of *T. leonina* (20 records) and *T. canis* (31 records) infections were 14.3% (95% CI: 8.1–22.0%) and 13.8% (95% CI: 9.8–18.3%), respectively (Figs. 2, 3 and 4 and Table 3). Necropsy was the method of choice for detection *Toxocara*/*Toxascaris* spp., implicating in 31.3% (95% CI: 20.6–43.0%) prevalence of *T. cati*, 18.8% (95% CI: 10.2–29.1%) frequency of *T. leonina*, and 17.2% (95% CI: 9.8–26.1%) prevalence of *T. canis* (Figs. 5, 6 and 7).

### Geographical characteristics

**Fig. 8:**
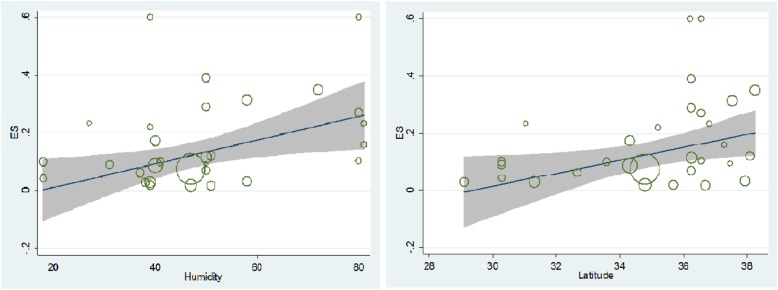
The meta-regression graph for the prevalence of *T. canis* according to humidity and latitude (*P* = 0.023), to (*P* = 0.032), respectively

There was no statistically significant association between the estimated pooled prevalence of *Toxocara*/*Toxascaris* infection in human population and mean temperature (*P* = 0.49), humidity (*P* = 0.49), longitude (*P* = 0.7), and latitude (*P* = 0.27). Among three parasite species, only humidity (*P* = 0.023) and latitude (*P* = 0.032) for *T. canis* were statistically significant, while others were not remarkably involved (Fig. 8).

## Discussion

**Fig. 9:**
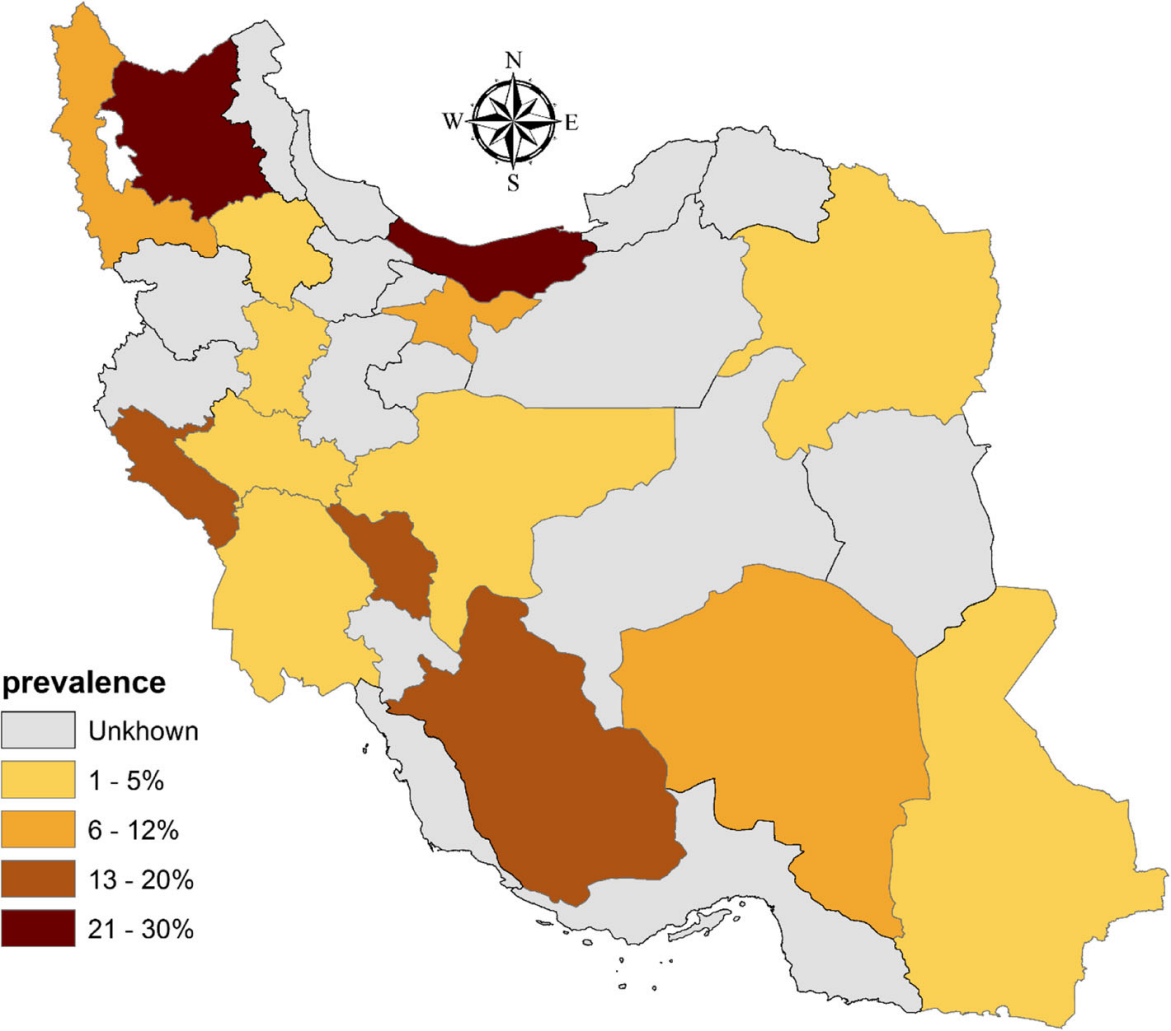
Distribution map of human *Toxocara*/*Toxascaris* prevalence by province in Iran

The current systematic review and meta-analysis was aimed to elucidate the prevalence of *Toxocara* spp. infection in animal and human hosts in Iran. The human infection was highly concentrated in two northern provinces (Mazandaran and East Azerbaijan) (Fig. 9), highlighting optimum geo-ecological milieu in those parts of the country because of high percentage humidity due to vicinity to the Caspian Sea as well as considerable rainfall during the year; notwithstanding, we didn’t found any statistically significant correlation between human *Toxocara*/*Toxascaris* seroprevalence studies and geographical parameters comprising mean temperature, humidity, longitude and latitude (Fig. 8). Despite of equal records of *Toxocara*/*Toxascaris* infection from rural and urban areas, seroprevalence was partly elevated in urban regions rather than rural territories, resulting from the likely heterogeneity among studies and/or lack of sufficient records; care must be taken in interpreting such result as rural areas are naturally considered as higher risk areas than urban [9, 11, 17, 20].

Toxocariasis due to several species of *Toxocara* and/or *Toxascaris* roundworms is still a seriously notifiable public health issue, particularly due to its intricate transmission routes [25]. In human this infection is caused by *T. canis*, in particular, and *T. cati* renders several issues comprising VLM, OLM, NLM and covert disease, each of which is represented by manifestations of the involved organ [26, 27]. *Toxocara*/*Toxascaris* infection in human populations is considered as a chronic parasite in nature which is distributed worldwide, particularly in tropical underdeveloped countries [28]. Several risk factors are supposed to play a major role in *Toxocara*/*Toxascaris* distribution among the human population, consisting of habitation in rustic areas, soil contact, consuming the undercooked meat of the infected paratenic host, insufficient and unhygienic water repositories, poor housing and low education as well [29–32]. Furthermore, owing to the adventurous nature of children, such as tasting any objects, eating soil and/or earthworms and being in the vicinity of dogs and cats, they are considered as a substantial risk group regarding toxocariasis [4, 33]. Hence, public places in which children may walk around such as parks, playgrounds, beaches and sandboxes are crucial territories for the acquisition of the infection [28, 31]. Since most individuals do not manifest any pathognomonic symptoms, the actual prevalence rate of the infection remains to be elucidated, even in industrialized nations [34, 35]. Considering that *Toxocara* parasites do not develop into adult stage in humans, coproscopy is unnecessary; thus, biopsy and direct parasite observation are the gold standard methods [36]. However, such examination is invasive and relies on the larval load and the infection phase [28]. Therefore, routine diagnosis of infection and/or exposure in human cases can be done by ELISA to detect specific antibody against TES antigens, which should be further validated by immunoblotting [37, 38]. As previously mentioned, TES-based ELISA tests are mostly used for human seroprevalence studies. Despite having proper immunogenicity, native TES antigens may cross-react with antibodies elicited against other helminths specifically *Ascaris lumbricoides* which decreases test specificity [39]. Therefore, the results may be regarded as suspicious, particularly when no immunoblotting confirmation is done, specifically in endemic regions where there exists the possibility of helminth co-infections. Alternative detection methods in paratenic or accidental hosts are including pathological inspection, larvae morphometry as well as PCR-based experiments [4]. A great deal of effort has been devoted to revealing the seroprevalence of human *Toxocara*/*Toxascaris* infection worldwide. In Africa, elevated seroprevalence rates of infection were detected, encompassing 6% in Egypt to 60% in Gabon and 92% in Réunion Island [5, 40]. Additionally, the seroprevalence ranges in Asia and South America included 11 -84.6% and 7.3–66%, respectively [41–43]. Comparable to other territories, rates of seropositive human cases were relatively low in European and North American countries [3], implicating improved hygiene practices and public awareness in industrialized nations.

In total, seroprevalence data integration in epidemiological investigations is not reasonable for several reasons, comprising sampling disparities, antigen preparation, and quality, different cutoff levels, cross-reactivity especially in the tropics were polyparasitism exist and inability to explicitly distinguish the infection by various *Toxocara* spp. Therefore, expanding our evidence based on human *Toxocara* infection would be corroborated by a better understanding of parasite biology, in particular, the immune evasion mechanism of larvae, and utilization of advanced, species-specific diagnostic tools [30].

The calculated total prevalence of infection in cats (*Felis catus*) was higher [32.6% (95% CI: 22.6–43.4%)] than in dogs (*Canis familiaris*) [24.2% (95% CI: 18–31%)] in the country (Table 1). Similar to human seropositive cases, carnivores in northern Iran were the most frequent hosts being parasitized by *Toxocara* spp., whereas minimum animals were infected in central parts [12% (95% CI: 8–17%)]. Among wild canine species in Iran, only jackal (*Canis aureus*) and red fox (*Vulpes vulpes*) were diagnosed with *Toxocara*/*Toxascaris* infection, with 23.3% (95% CI: 7.7–43.2%) and 69.4% (95% CI: 60.3–77.8%), respectively (Table 1).

Moreover, it was deduced that the weighted prevalence of *T. canis*, *T. cati*, and *T. leonina* in Iran were 13.8% (95% CI: 9.8–18.3%), 28.5% (95% CI: 20.0–37.7%), and 14.3% (95% CI: 8.1–22.0%), respectively. Given geographical characteristics, only humidity (*P* = 0.023) and latitude (*P* = 0.032) were significantly linked to *T. canis* infection. Increasing latitude would likely result in decreased mean temperature and more temperate climates than the equator area. Water vaporization and condensation in northern parts of the country due to the vicinity to the Caspian Sea and high mountain ranges and humid weather substantially implicate in *Toxocara*/*Toxascaris* larval development, as proved in the laboratory [17, 20].

The survey of the infection in carnivores is usually made via traditional parasitological methods (e.g. floatation technique) to detect eggs as well as intestinal necropsy of dead carcasses [44, 45]. Nevertheless, each detection method may provide a prevalence rate different from other modalities, which this issue would implicate potential biases in reporting and/or interpreting data. As we stated in the results section, necropsy has been shown as a better and efficient detection tool than fecal examination. For instance, more than 2-fold prevalence of *Toxocara*/*Toxascaris* spp. in dogs was obtained using necropsy [34.3% (95% CI = 26.4–42.8%)] than fecal examination [15.6% (95% CI = 9.8–22.4%)]. Also, necropsy was the most efficacious method in cats with 37.4% (95% CI = 23.5–52.4%) than fecal examination [20.4% (95% CI = 9.4–34.2%)]. On an international scale, different studies have documented the prevalence of *Toxocara*/*Toxascaris* in stray/domestic dogs (*Canis familiaris*) and cats (*Felis catus*). In Europe, *T. canis* prevalence in dogs ranged from 1% in Germany to 76% in Albania. Also, the prevalence of *T. cati* infection was up to 34.5% in Spain in this continent [46–49]. In dogs dwelling in the Americas, *T. canis* infection prevalence varied from 12.7% in Canadian provinces to 18% in Cuba. Also, *T. cati* was mostly prevailed in Argentina and Brazil with 61 and 25%, respectively [50–53]. The highest *T. canis* and *T. cati* infection rates in Asia were dedicated to Russia and China with 63 and 36.5%, respectively [54, 55]. Additionally, mild *Toxocara* species infections were identified in African domestic carnivores [56–59].

Globally, the highest *T. leonina* prevalence (up to 38%) was observed in domestic dogs from Russia [52]. Wildlife probably plays a critical role in the epidemiology of *Toxocara* species, as they may be considered as potent reservoir for these enigmatic roundworms [60].

Patent *T. canis* infections are generally higher in young foxes (under 6 months of age); although, a relatively high prevalence rate have also been among adult foxes in endemic territories, representing weak immune status against intestinal [61]. The prevalence of *T. canis* in European foxes varies between 9.0% (in Italy) and 65.0% (in Denmark), as well as 32.5 and 71.0% prevalence in Canada and Japan, respectively [61]. The lowest and highest *T. leonina* prevalence in red fox was reported from Kirghizstan (5.9%) and the Slovak Republic (47.1%), respectively [60]. Regarding golden jackal (*Canis aureus*) moderate prevalence rates of *Toxascaris leonina* have been reported around the world, such as in Azerbaijan (31.8%), Bulgaria (36%) and Russia (43.5%). The prevalence of *T. canis* in this wildlife species ranges 40–61% in Asia and 20–54.5% in European countries, whereas *T. cati* was only detected in jackals dwelling in Russia (5–26%) [49, 62]. Considering that there are only 4 golden jackal studies and 2 red fox (*Vulpes vulpes*) studies, there exist paucity of data on *Toxocara*/*Toxascaris* prevalence in wild canine and feline fauna of Iran, which highlights more subtle investigations. Approximately, since the middle of previous century a periurban rise in European foxes population carrying *Toxocara*/*Toxascaris* worm burdens have posed a great environmental risk of contamination with parasite ova. On the other hand, they act a critical role in maintaining *T. canis* wildlife cycle with implications in constant transmission to human populations and pet dogs [63].

The findings of the present study indicated a mild seroprevalence in human population; also, infection in cats was higher than dogs, however unbalanced sampling may have influenced these findings. Most of the infected cases were from north of Iran, which possess a favorable ecological milieu for appropriate animal hosts and *Toxocara* egg development (i.e., 28–33 °C in laboratory-based conditions, during 2–6 weeks [64]. Despite the improved hygiene and health surveillance systems as well as a wide-range public awareness in developed countries, still *Toxocara*/*Toxascaris* infection remains a public health concern in those areas and the rest of the world as well. During the time, there have been established a close companionship between dogs and cats with humans, and during past decades it has been even strengthened. However, these associations, particularly in underdeveloped nations, have been accompanied with poor veterinary infrastructures. This, along with free-roaming or community-owned dogs and cats pose a serious threat for zoonoses transmission to human societies [65].

With respect to the constant infection cycle in carnivores and the life-threatening traits of human toxocariasis, revisiting the epidemiological strategies in companion animals enclosing anti-helminthic medication and screening plans such as the routine fecal examination is of utmost importance. In addition, it is highly emphasized that future human investigations focus on using recombinant TES antigens with high sensitivity and specificity and less cross-reactivity. Also, it is better to identify anti-*Toxocara* IgG_4_ coupled with TES rather than total IgG and employ western blot as a complementary diagnostic technique [28]. Moreover, it is recommended to educate laboratory technicians for accurate parasite detection, regularly deworm puppies and kittens to decrease the worm burden, perform pro-active chemoprophylaxis approach and cultivate knowledge among the public as well as physicians regarding the clinical consequences of the disease. The interwoven collaboration among blood banks, veterinary diagnostic laboratories and municipalities (control stray dog/cat populations in urban areas) would provide a more completed picture of disease seroprevalence and distribution in people and animals, giving us the opportunity for targeted intervention strategies and better management of this zoonotic enigma. In parallel to above-mentioned recommendations the WSAVA has recently found a One Health Committee to highlight the transmission potential of zoonotic infectious agents from dog/cat to human. Besides the OIE has recently extended the surveillance of wildlife diseases through WAHID in the world. All of these expanded fields of epidemilogical data would assist the global community towards better understanding of human-domestic animal-wildlife interplay and control of human zoonotic diseases [63].

## Limitations

It is noteworthy to mention that some limitations constrained our findings en route performing current systematic review and meta-analysis, including 1) lack of risk factor appraisal, 2) absence of a standard, easy-to-use diagnostic tool in case of human studies to particularly discern the involved *Toxocara* spp., 3) lack of investigations considering different aspects of human *Toxocara*-induced complications such as VLM, OLM, and covert infection. Certainly, with these in hands, we could achieve the more complete picture of the current situation of *Toxocara/Toxascaris* infection in animal and human hosts of Iran.

## Conclusion

In conclusion, this study revealed that *Toxocara* and *Toxascaris* infection in Iran among people is mild while in dogs and cats are high. Exclusive studies including human, animal and environmental health data should be conducted in different geographical regions of the country. The outcome of such studies will allow the government and non-government organization to set proprieties and design strategies, combining accurate surveillance and prevention of these zoonotic diseases.

## Supplementary information

**Additional file 1: Figure S1.** The quality assessment of included studies of human population

**Additional file 2: Figure S2.** The weighted prevalence of human *Toxocara*/*Toxascaris* by the year in Iran

**Additional file 3: Figure S3.** The weighted prevalence of human *Toxocara*/*Toxascaris*by the age in Iran

**Additional file 4: Figure S4.** The weighted prevalence of *Toxocara*/*Toxascaris* in Iran dogs by study method

**Additional file 5: Figure S5.** The total prevalence of *T. canis* in feces of animals according to the different parasitology methodsin carnivore population in Iran.

**Additional file 6: Figure S6.** The total prevalence of *T. cati* in feces according to the different parasitology methods in carnivore population in Iran

## Data Availability

The datasets used and analyzed during the current study are available from the corresponding author on reasonable request.

## Abbreviations

CI: Confidence interval
ELISA: Enzyme-linked immunosorbent assay
IgE: Immunoglobulin E
IgG: Immunoglobulin G
IL: Interleukin
JBI: Joanna Briggs Institute
NLM: Neural larva migrans
OIE: Organization for Animal Health
OLM: Ocular larva migrans
PCR: Polymerase chain reaction
SID: Scientific information database
STATA: Statistics and data
*T. canis*: *Toxocara canis*
*T. cati*: *Toxocara cati*
*T. leonina*: *Toxascaris leonina*
TES: *Toxocara* excretory-secretory
Th: T-helper
VLM: Visceral larva migrans
WAHID: World Animal Health Information Database
WSAVA: World Small Animal Veterinary Association
