# Supplementary figures and images for: Prevalence of Toxocara and Toxascaris infection among human and animals in Iran with meta-analysis approach
Source: BMC Infect Dis. 2020 Jan 7;20:20. doi: 10.1186/s12879-020-4759-8 (PMC6947998; doi:10.1186/s12879-020-4759-8)

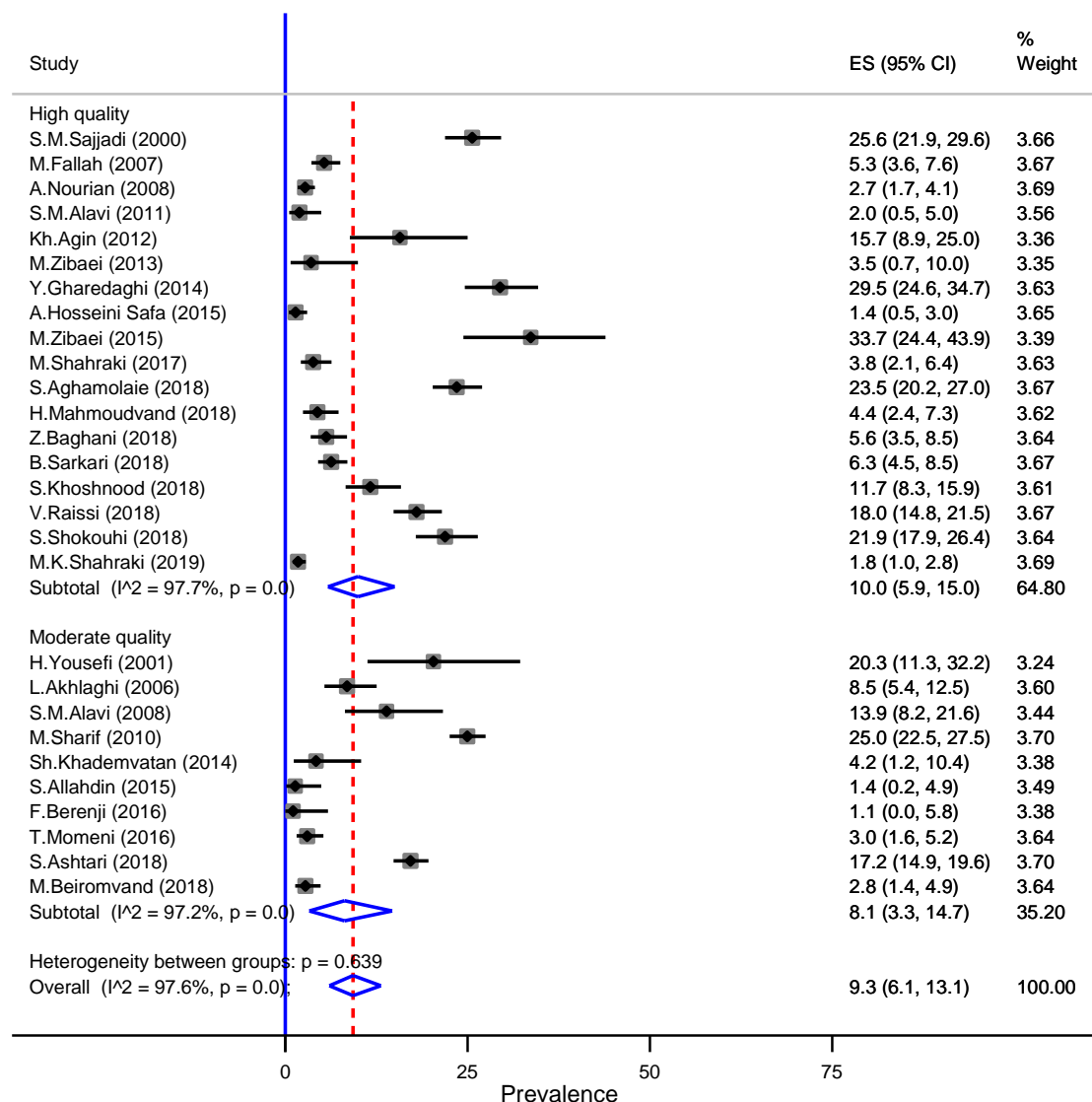

**Supplementary Fig. 1** The quality assessment of included studies of human population

Supplement: Supplementary file 1 — Additional file 1: Figure S1. The quality assessment of included studies of human population [file 12879_2020_4759_MOESM1_ESM.pdf]

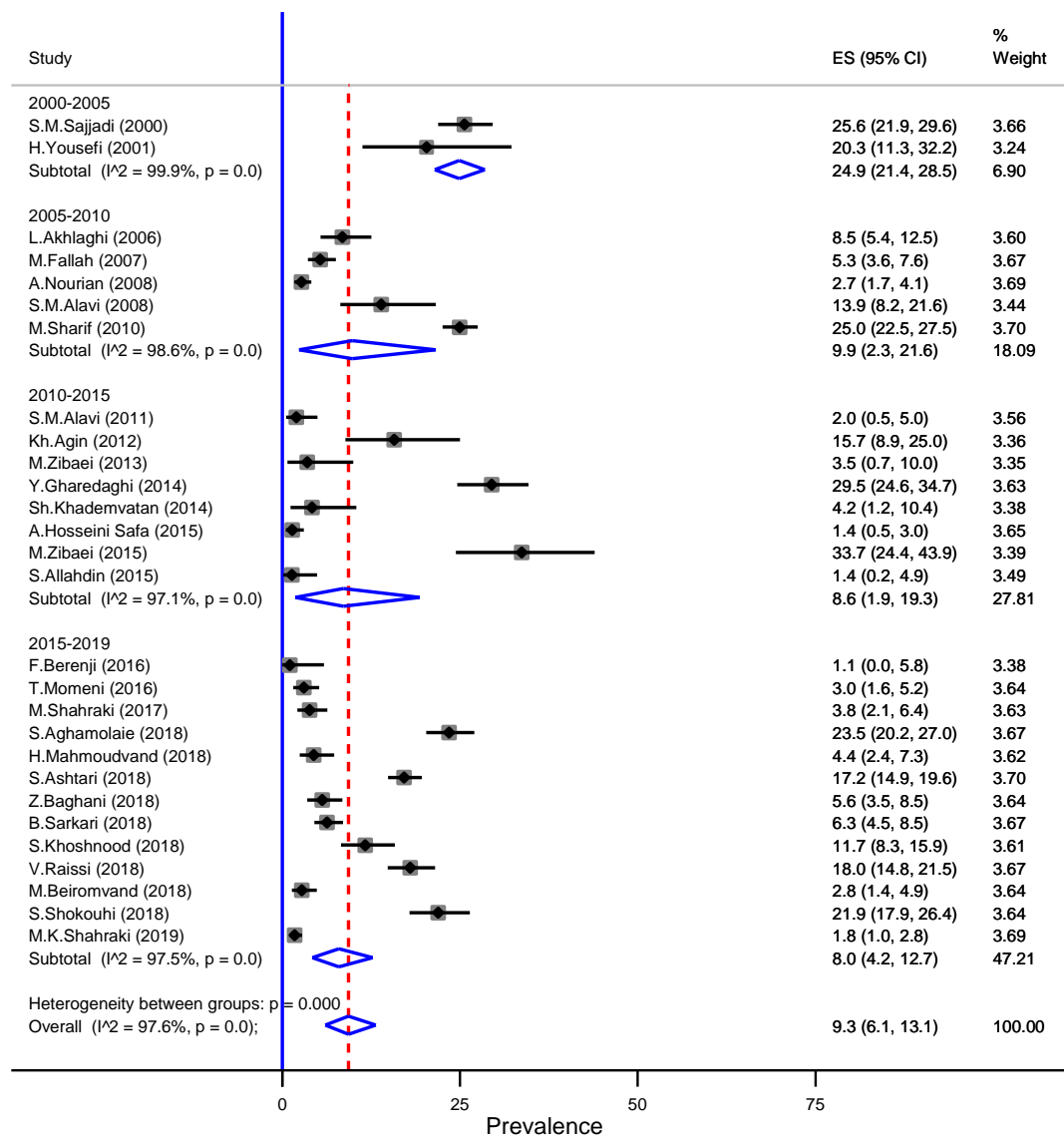

**Supplementary Fig. 2** The weighted prevalence of human *Toxocara/Toxascaris* by the year in Iran

Supplement: Supplementary file 2 — Additional file 2: Figure S2. The weighted prevalence of human Toxocara/Toxascaris by the year in Iran [file 12879_2020_4759_MOESM2_ESM.pdf]

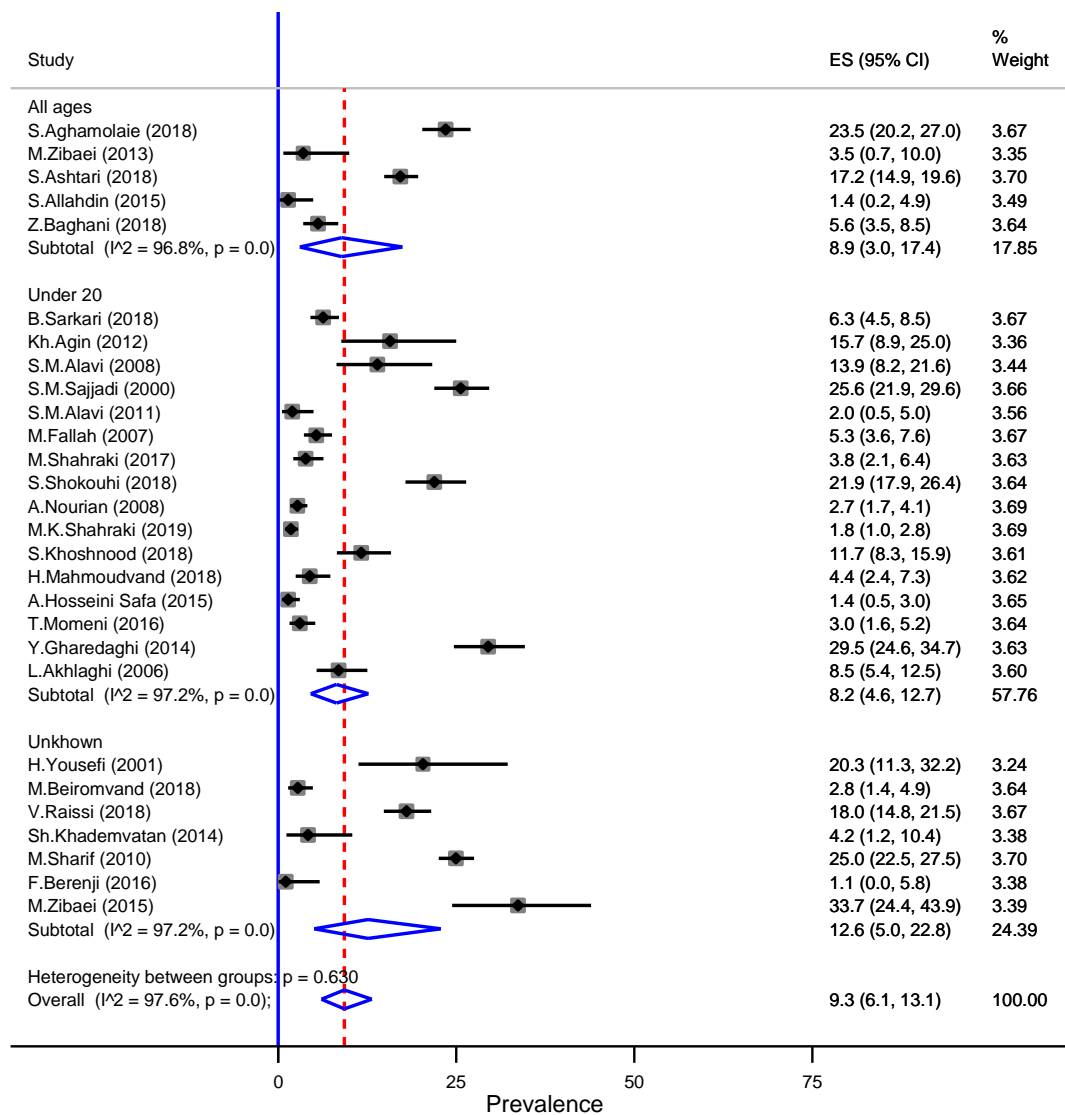

**Supplementary Fig. 3** The weighted prevalence of human *Toxocara/Toxascaris* by the age in Iran

Supplement: Supplementary file 3 — Additional file 3: Figure S3. The weighted prevalence of human Toxocara/Toxascarisby the age in Iran [file 12879_2020_4759_MOESM3_ESM.pdf]
