# Supplementary material for: Prevalence of Toxocara and Toxascaris infection among human and animals in Iran with meta-analysis approach
Source: BMC Infect Dis. 2020 Jan 7;20:20. doi: 10.1186/s12879-020-4759-8 (PMC6947998; doi:10.1186/s12879-020-4759-8)
Supplement: Supplementary file 4 — Additional file 4: Figure S4. The weighted prevalence of Toxocara/Toxascaris in Iran dogs by study method [file 12879_2020_4759_MOESM4_ESM.pdf]

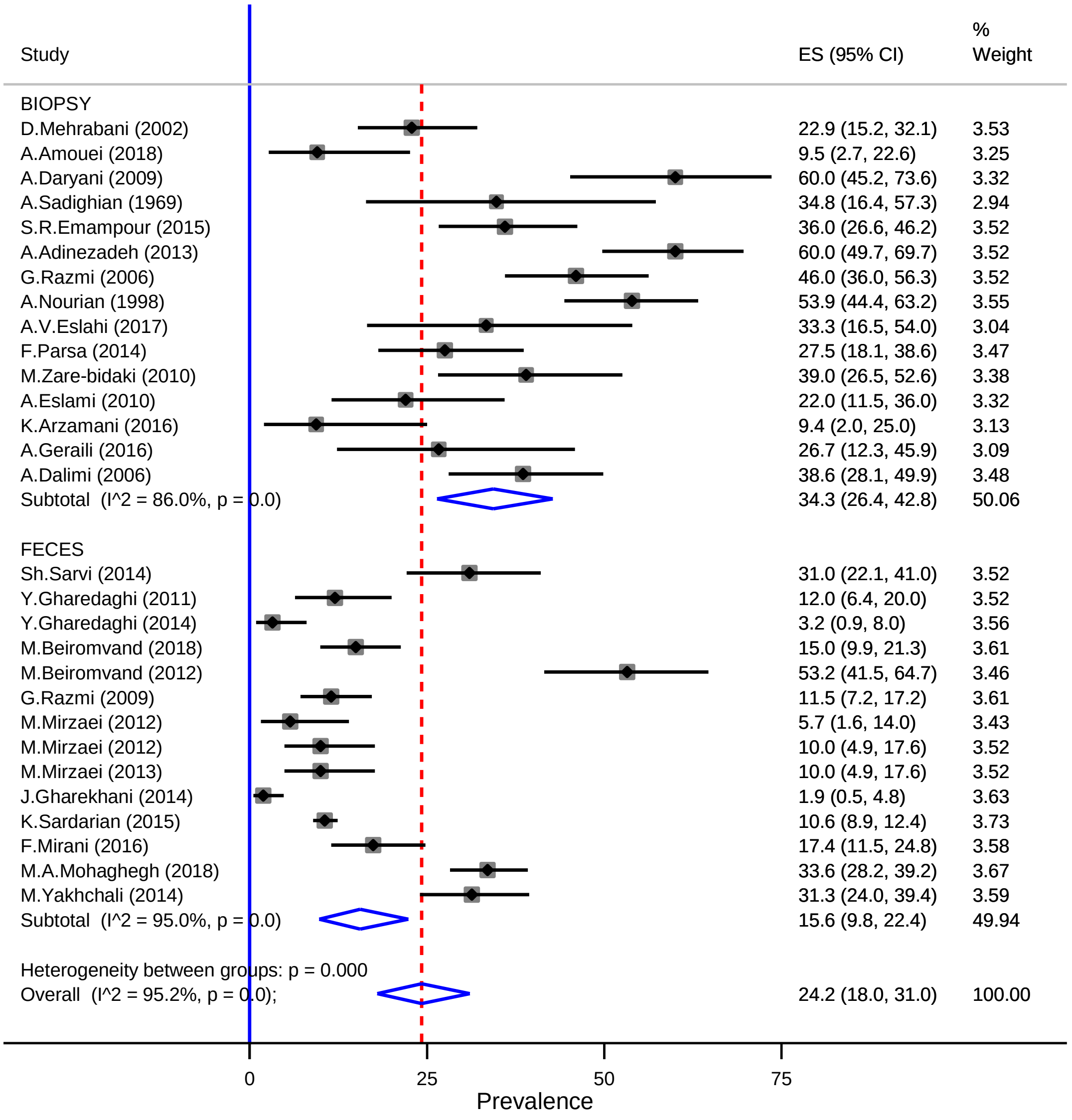

Supplementary Fig. 4 The weighted prevalence of *Toxocara*/*Toxascaris* in Iran dogs by study method
