# Supplementary material for: Prevalence of Toxocara and Toxascaris infection among human and animals in Iran with meta-analysis approach
Source: BMC Infect Dis. 2020 Jan 7;20:20. doi: 10.1186/s12879-020-4759-8 (PMC6947998; doi:10.1186/s12879-020-4759-8)
Supplement: Supplementary file 5 — Additional file 5: Figure S5. The total prevalence of T. canis in feces of animals according to the different parasitology methodsin carnivore population in Iran. [file 12879_2020_4759_MOESM5_ESM.pdf]

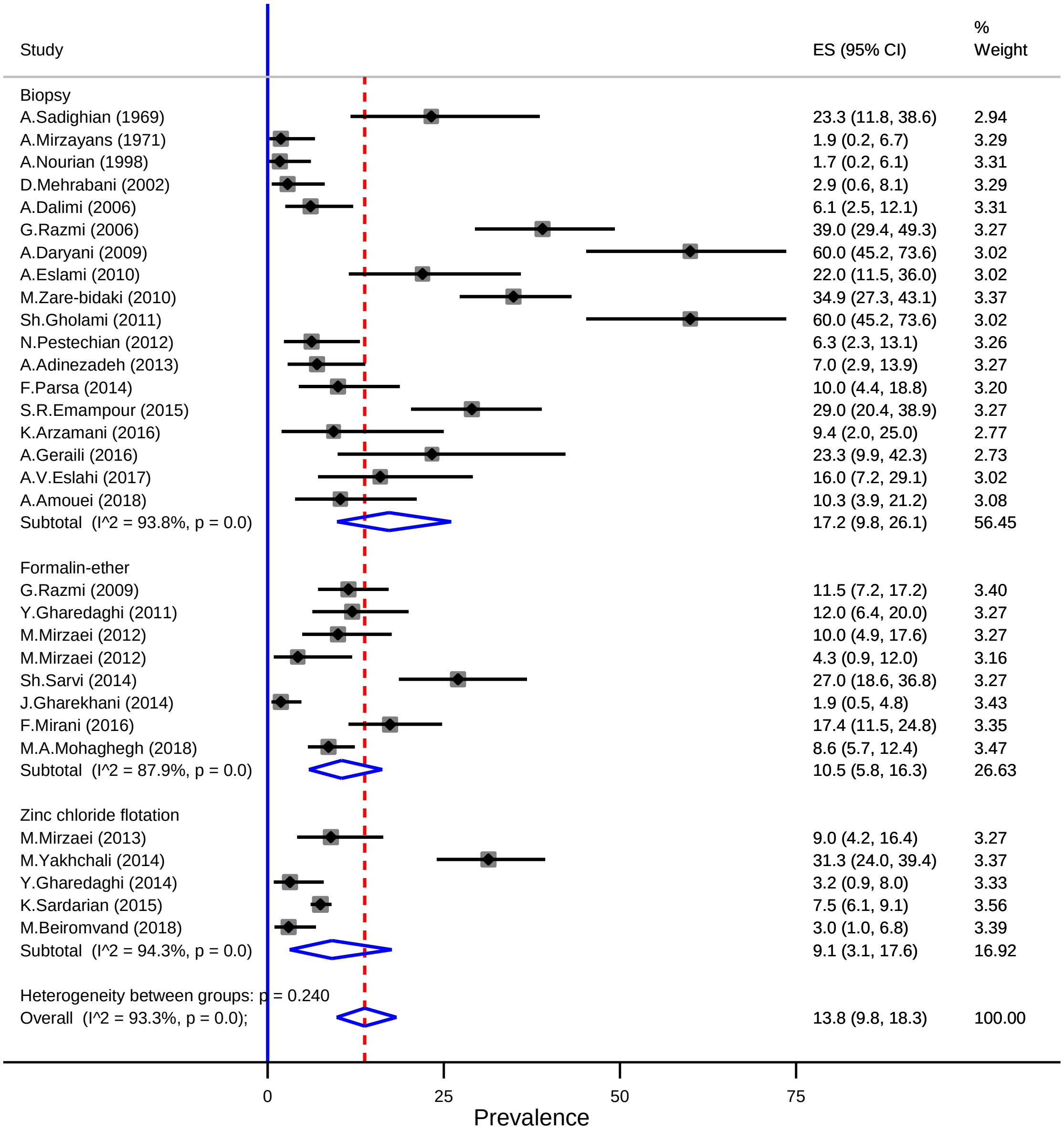

Supplementary Fig. 5 The total prevalence of *T. canis* in feces of animals according to the different parasitology methodsin carnivore population in Iran
