# Supplementary material for: Prevalence of Toxocara and Toxascaris infection among human and animals in Iran with meta-analysis approach
Source: BMC Infect Dis. 2020 Jan 7;20:20. doi: 10.1186/s12879-020-4759-8 (PMC6947998; doi:10.1186/s12879-020-4759-8)
Supplement: Supplementary file 6 — Additional file 6: Figure S6. The total prevalence of T. cati in feces according to the different parasitology methods in carnivore population in Iran [file 12879_2020_4759_MOESM6_ESM.pdf]

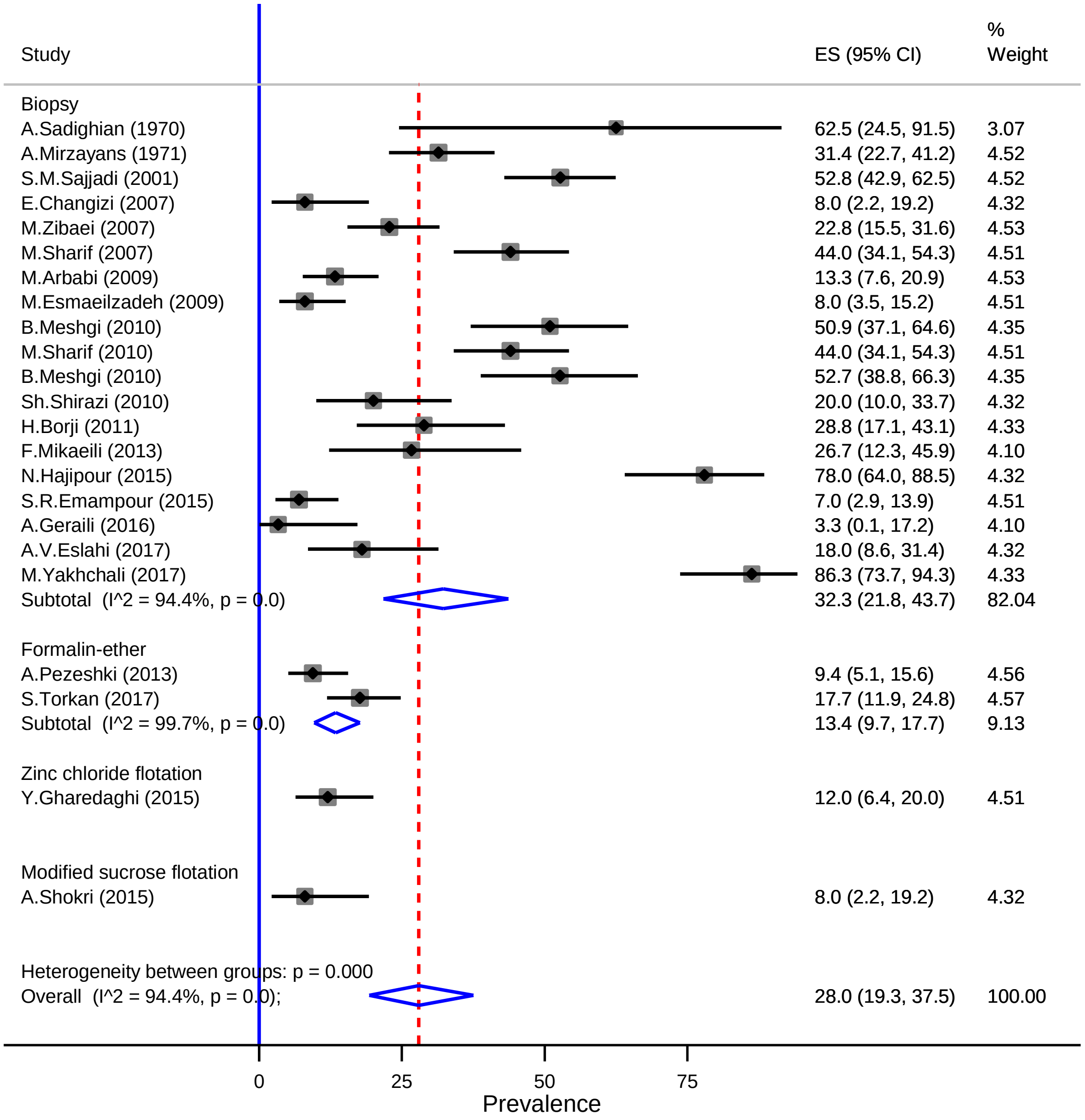

Supplementary Fig. 6 The total prevalence of *T. cati* in feces according to the different parasitology methods in carnivore population in Iran
